# Supplementary material for: Comparative Transcriptomics Identify Key Hypothalamic Circular RNAs that Participate in Sheep (Ovis aries) Reproduction
Source: Animals (Basel). 2019 Aug 14;9(8):557. doi: 10.3390/ani9080557 (PMC6721059; doi:10.3390/ani9080557)
Supplement: Supplementary file 1 [file animals-09-00557-s001.zip › Additional files/Legend.docx]

**Additional files**

**Table S1.** Real-time quantitative polymerase chain reaction primers and sizes of the amplification products of the selected circular RNAs and housekeeping genes.

**Table S2.** Overview of the quality control of mRNA reads generated from hypothalamic tissues. **Table S3.** All circular RNAs identified in hypothalamic tissues.

**Table S4.** All circular RNAs identified in polytocous sheep in polytocous sheep in follicular phase versus monotocous sheep in follicular sheep (PF vs. MF), and in polytocous sheep in luteal phase versus monotocous sheep in luteal phase (PL vs. ML), where yellow represents differentially expressed circular RNAs.

**Table S5.** GO enrichment annotation for host genes of differentially expressed circular RNAs in terms of the categories of molecular function (MF), biological process (BP), and cellular component (CC) in polytocous sheep in follicular phase versus monotocous sheep in follicular phase (PF vs. MF), and polytocous sheep in luteal phase versus monotocous sheep in luteal phase (PL vs. ML).

**Table S6.** KEGG enrichment annotation for host genes of differentially expressed circular RNAs in polytocous sheep in follicular phase versus monotocous sheep in follicular sheep (PF vs. MF), and polytocous sheep in luteal phase versus monotocous sheep in luteal phase (PL vs. ML).

**Table S7.** A list of all circular RNA–microRNA pairs predicted by miRanda databases.

**Table S8.** Target genes of oar-miR-665-3p predicted by TargetScan database.
